# Management of inappropriate sexual behaviour in frontotemporal dementia: a case study

**DOI:** 10.1192/bjo.2021.343

**Published:** 2021-06-18

**Authors:** Stefan McKenzie, Zoe Kwan, Velusamy Sivakumar

**Affiliations:** Sheffield Health and Social Care NHS Foundation Trust

## Abstract

**Objective:**

To present a case of a 79-year-old male with frontal lobe dementia (following a cerebral abscess) who was referred due to inappropriate sexualised behaviour (ISB) in a care home setting.

To discuss the evidence base for the management of ISB in frontotemporal dementia.

**Case report:**

79-year-old male patient who was diagnosed with frontal lobe dementia, following a craniotomy to aspirate and evacuate a cerebral abscess which affected the left frontal, parietal and temporal lobes. He then started to exhibit sexualised behaviour; he was using sexualised language towards female residents and care workers in the residential home, and was inviting residents to his room and asking them to touch him. This behaviour was felt to be due to inappropriate sexual behaviour which forms part of the spectrum of behavioural and psychological symptoms of dementia. Non-pharmacological interventions were tried but failed to manage his symptoms. He was started on Paroxetine which treated the symptoms for approximately 12 months. The symptoms reocurred and he was switched to Amisulpride which had a positive effect on his symptoms.

**Discussion:**

ISB is a behavioural and psychological symptom of dementia and may be seen in 7% to 25% of patients with dementia. ISB is distressing for the caregivers and also presents considerable challenges for the treating clinician. ISB presents with behaviour such as sexual language, implied sexual acts, and overt sexual acts. A differentiation should be made between whether the act was one of intimacy-seeking or disinhibition. However, there is a need to intervene when there are risks to the wellbeing and safeguards of the patient and also caregivers and residents. ISB can be difficult to treat, and there is limited evidence on the subject. It is often better managed by non-pharmacological interventions if possible, due to patients often being less responsive to psychoactive therapies and the risks involved with using medication. Non-pharmacological interventions include environmental, behavioural and educational approaches, and examples of these are discussed. Pharmacological interventions are also discussed, but there is a lack of evidence in this area; currently the evidence is from case series and case reports. The variety of drug classes illustrate the non specific nature of drug therapy.

**Conclusion:**

Managing and treating ISB is difficult and complex.

The evidence suggests using non-pharmacological approaches as first line before considering pharmacological interventions.

However, there is a need for further research to develop robust non-pharmacological and pharmacological interventions in the treatment of ISB.

